# Dual-ratio approach to pulse oximetry and the effect of skin tone

**DOI:** 10.1117/1.JBO.29.S3.S33311

**Published:** 2024-10-12

**Authors:** Giles Blaney, Jodee Frias, Fatemeh Tavakoli, Angelo Sassaroli, Sergio Fantini

**Affiliations:** Tufts University, Department of Biomedical Engineering, Medford, Massachusetts, United States

**Keywords:** optical pulse oximetry, blood oxygen saturation, near-infrared spectroscopy, melanin, hemodynamics, dual ratio

## Abstract

**Significance:**

Pulsatile blood oxygen saturation (${\mathrm{SpO}}_{2}$) via pulse oximetry is a valuable clinical metric for assessing oxygen delivery. Individual anatomical features, including skin tone, may affect current optical pulse oximetry methods.

**Aim:**

We developed an optical pulse oximetry method based on dual-ratio (DR) measurements to suppress individual anatomical confounds on ${\mathrm{SpO}}_{2}$.

**Approach:**

We designed a DR-based finger pulse oximeter, hypothesizing that DR would suppress confounds from optical coupling and superficial tissue absorption. This method is tested using Monte Carlo simulations and *in vivo* experiments.

**Results:**

Different melanosome volume fractions in the epidermis, a surrogate for skin tone, cause changes in the recovered ${\mathrm{SpO}}_{2}$ on the order of 1% in simulation and *in vivo*. Different heterogeneous pulsatile hemodynamics cause greater changes on the order of 10% in simulations. ${\mathrm{SpO}}_{2}$ recovered with DR measurements showed less variability than the traditional single-distance (SD) transmission method.

**Conclusions:**

For the models and methods considered here, ${\mathrm{SpO}}_{2}$ measurements are strongly impacted by heterogeneous pulsatile hemodynamics. This variability may be larger than the skin tone bias, which is a known confound in ${\mathrm{SpO}}_{2}$ measurements. The partial suppression of variability in the ${\mathrm{SpO}}_{2}$ recovered by DR suggests the promise of DR for pulse oximetry.

## 1 Introduction

Pulse oximetry allows for the non-invasive measurement of pulsatile blood oxygen saturation (${\mathrm{SpO}}_{2}$) (The “p” in ${\mathrm{SpO}}_{2}$ is defined as “pulsatile” here to represent the origin of the signal. However, others have also donated the “p” as meaning “peripheral” or “perfusion.”) [i.e., a surrogate for arterial blood oxygen saturation (${\mathrm{SaO}}_{2}$)] in various clinical settings.^1^^–^^5^ ${\mathrm{SpO}}_{2}$ measurements by pulse oximetry have become ubiquitous in modern healthcare, providing valuable real-time assessment of patients’ oxygen delivery. The history of pulse oximetry may be considered to start with Glenn Millikan, who invented the first practical oximeter in the 1940s.^1^^,^^6^^,^^7^ This invention was followed by Takuo Aoyagi’s next technological advance in the 1970s when they developed pulse oximetry into something similar to today’s technology.^2^ However, despite pulse oximetry’s widespread adoption and long history, open questions still exist regarding how differences between different people, such as skin tone, would confound the recovered ${\mathrm{SpO}}_{2}$.^8^^–^^16^ These questions open the door for modern investigations of the technique and the proposal of novel oximetry methods.

Various recent publications have focused on the impact of skin tone on ${\mathrm{SpO}}_{2}$ readings by pulse oximetry. A recent letter examining ${\mathrm{SpO}}_{2}$ versus ${\mathrm{SaO}}_{2}$ on a large population of patients found a positive bias in ${\mathrm{SpO}}_{2}$ for Black versus White patients (i.e., the true ${\mathrm{SaO}}_{2}$ was lower on average for Black patients compared with White who showed the same ${\mathrm{SpO}}_{2}$ reading).^17^ Furthermore, other recent studies, reviews, and meta-analyses suggest a similar bias.^10^^–^^12^^,^^14^ In these cases, the bias is on the order of a few percent on ${\mathrm{SpO}}_{2}$, but this bias may become more pronounced at lower ${\mathrm{SaO}}_{2}$.^8^

The measurement of ${\mathrm{SpO}}_{2}$ relies on the ratio of pulsatile optical signals at red and infrared wavelengths. Some work has identified a possible issue with the broad spectral bandwidth of light-emitting diodes (LEDs) when they are used as the optical source.^18^^,^^19^ However, to alleviate this concern, we consider single-wavelength sources, which is a good assumption for the laser diodes (LDs) in this work. A physiological confound may arise from characteristics of the pulsatile hemodynamics, for example, low perfusion resulting in a small pulsatile amplitude.^20^ Aside from confounds of an instrumental or physiological origin, further recent studies seek to understand the possible origins of these biases in terms of diffuse light transport and investigate ways to mitigate them by modeling the pulse oximetry measurements using methods such as Monte Carlo (MC) simulations. One such study modeled the effect of the volume fraction of melanosomes ([M]) in the epidermis and found it had a slight effect on ${\mathrm{SpO}}_{2}$, with [M] primarily impacting the average total optical path length (⟨L⟩) and the measured intensity (I).^21^ A second study utilized similar methods but focused more directly on the effect of [M] on ${\mathrm{SpO}}_{2}$ calibration, finding a positive bias for darker skin tones around 1% to 2%.^16^ The biomedical optics field is actively investigating the observed skin tone bias on ${\mathrm{SpO}}_{2}$ measurements, but a definite consensus has yet to be reached. The lack of definite consensus may suggest that there are differences between persons with different skin tones that have yet to be fully captured with current models. Furthermore, new pulse oximetry methods that will reduce this bias in the optical measurement itself have yet to be proposed. Such novel methods are needed to make pulse oximetry a valid clinical tool for all patients regardless of patient differences, such as skin tone.

In this work, we contribute to the investigation of the effect of skin tone on ${\mathrm{SpO}}_{2}$ measurements and propose a novel pulse oximetry method based on the dual-ratio (DR) technique. We focus on the diffuse light transport within the human finger given the presence of melanin and the effect of pulsatile hemodynamics with different amplitudes and phases. Our discussion is based on MC simulations and *in vivo* measurements of ${\mathrm{SpO}}_{2}$ on healthy human subjects. The MC model allows us to investigate the effect of the [M] in the epidermis on pulse oximetry measurements given either homogeneous or heterogeneous pulsatile hemodynamics. The novel component is the DR measurement type applied to the human finger. This geometry was inspired by a DR method we developed for measuring the absolute optical properties of turbid media in a cuvette.^22^ DR^22^^,^^23^ is a measurement technique based on the previously developed dual-slope (DS)^24^^,^^25^ and self-calibrating^26^ techniques. These techniques are insensitive to coupling changes and have suppressed sensitivity to local absorption change (S) near the optodes. These features of DR have the potential to make DR advantageous for ${\mathrm{SpO}}_{2}$ measurements as a result of the small sensitivity to skin-to-optode coupling or dynamics present in superficial tissue (i.e., the epidermis). Our rationale for proposing a DR-based technique is that confounds resulting in a skin tone bias are likely related to sensitivity to the epidermis, so a measurement type less sensitive to the epidermis should be less affected by these confounds and may have less bias. DR’s reduced sensitivity to superficial tissue is inherited from DS (If an optode arrangement can form both a DR and DS set, both DR and DS have the same region of S). Both methods rely on a symmetric arrangement of short and long source-detector distances (ρs), where the data from short distances is subtracted from that of the long. S maps for DS have been previously reported showing this feature of reduced S to superficial regions.^27^ In the following sections, we combine MC models with *in vivo* data to investigate [M]- or pulsatile-hemodynamic-heterogeneity-based confounds on ${\mathrm{SpO}}_{2}$. This is in the context of the extent to which those confounds affect either traditional single-distance (SD) measurements in transmittance or the novel DR measurement type applied to finger pulse oximetry.

## 2 Methods

Methods for this work can be divided into two categories: MC simulations (Sec. 2.3) and *in vivo* experimental measurements (Sec. 2.4). Outputs from the MC simulations were used to analyze and interpret the experimental *in vivo* data. We start by describing the measurement geometry (Sec. 2.1) and measurement types (Sec. 2.2) which are common to both.

### 2.1 Measurement Geometry

**Fig. 1:**
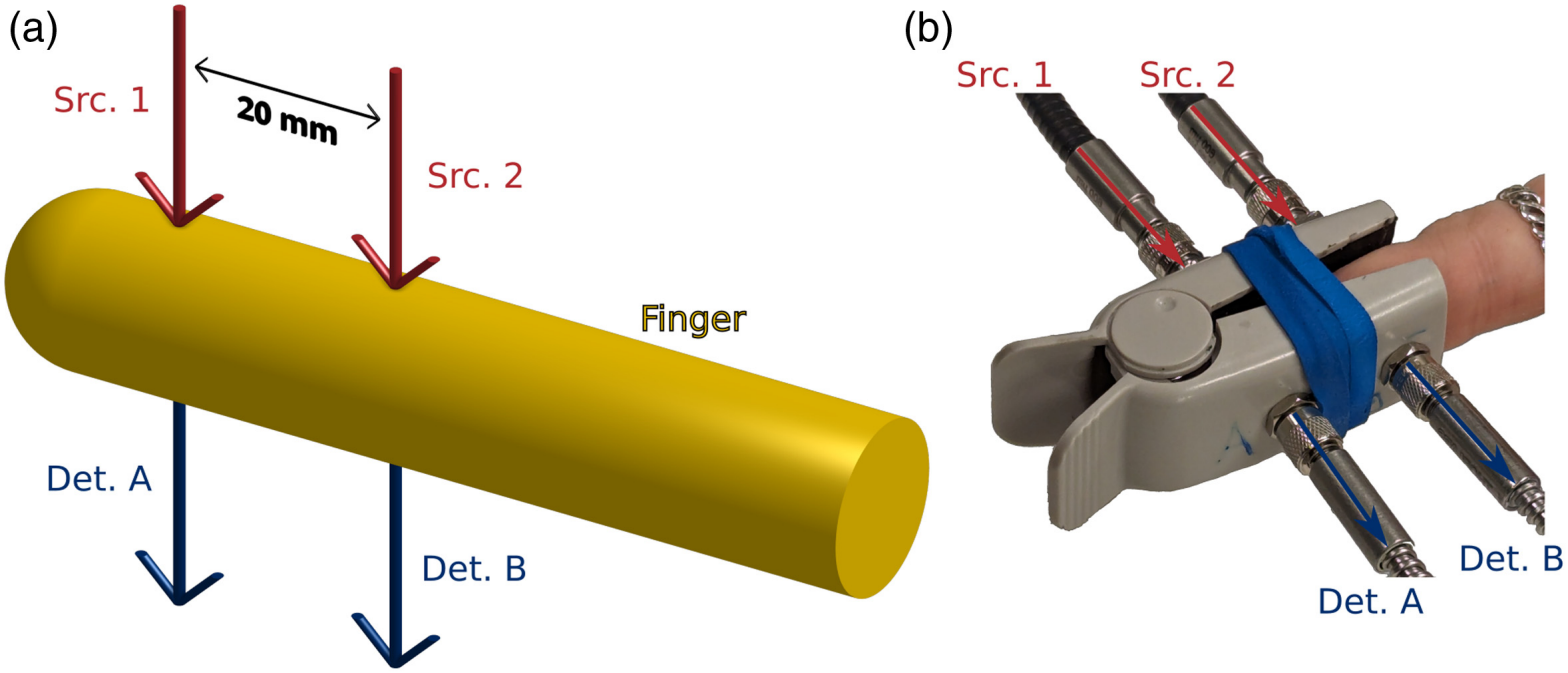
(a) Schematic of the measurement geometry. Two sources (Src.; 1 and 2) and two detectors (Det.; A and B) were utilized in a transmission geometry through the finger to achieve a DR set. Data were collected from all four possible SD source-detector pairs (i.e., 1A, 1B, 2A, 2B). Src. 1/Det. A and Src. 2/Det. B were spaced 20-mm apart. (b) Photo of the probe which utilized four optical fibers (i.e., one for each optode) and a standard pulse-oximeter finger clip. The finger was oriented, so the sources were on the nail/knuckle side, and Src. 1 was placed behind the nail such that light did not enter the finger through the nail.

We considered the geometry in Fig. 1 for all measurements and simulations in this work. Two source locations and two detector locations were used in a transmission geometry. Sources were named using numbers (1 and 2), whereas detectors were named using letters (A and B). As shown in Fig. 1(a), detector A was placed in line (i.e., in transmission) with source 1, and similarly, detector B was in line with source 2. The spacing between sources 1 and 2 was 20 mm, as shown in Fig. 1(a). Because corresponding sources and detectors were in-line, detectors A and B were also spaced by the same 20 mm. Figure 1(b) is a photo of the real-life measurement setup which realizes Fig. 1(a) using optical fibers and a modified pulse oximetry finger clip.

### 2.2 Measurement Types

Two types of measurement are considered in this work, SD and DR. SD is the traditional measurement utilized in near-infrared spectroscopy (NIRS) which is based upon the changes (i.e., with respect to baseline) of the natural logarithm of I measured between one source and one detector. Furthermore, the SD approach is fundamentally the same as the ratio of ratio approach currently used in pulse oximetry (which, in fact, relies on data collected at a single-source detector distance for each wavelength), differing only in the assumed calibration. These SD data may be converted to an effective absorption coefficient change ($\Delta \mu_{a}$) using the ⟨L⟩ (If one wishes to consider the ρ and the differential path length dactor (DPF) instead, we can write ⟨L⟩=ρDPF.) according to the following equation

$$\Delta \mu_{a,\mathrm{SD}}=-\frac{\ln[I]-\ln[I_{0}]}{\left\langle L\right\rangle },$$ (1)

where $I_{0}$ is the baseline Intensity and the subscript SD signifies that the quantity is an effective recovered $\Delta \mu_{a}$ from the SD measurement type. For the measurement geometry in this work (Fig. 1), there are four SDs (i.e., 1A, 1B, 2A, and 2B); however, for the results in this work, we focus on 1A.

To show that SD is effectively the same as current pulse oximetry methods, consider how ${\mathrm{SpO}}_{2}$ may be calculated^28^

$${\mathrm{SpO}}_{2}=\frac{a+b\{(\Delta I_{\mathrm{HR}}/I_{0}{)}_{\lambda_{1}}/(\Delta I_{\mathrm{HR}}/I_{0}{)}_{\lambda_{2}}\}}{c+d\{(\Delta I_{\mathrm{HR}}/I_{0}{)}_{\lambda_{1}}/(\Delta I_{\mathrm{HR}}/I_{0}{)}_{\lambda_{2}}\}},$$ (2)

where $\Delta I_{\mathrm{HR}}$ is the change from pulsatile variations at heart rate and a, b, c, and d are constants. Equation (2) is commonly linearized (i.e., by Taylor expansion), resulting in the form ${\mathrm{SpO}}_{2}=\alpha +\beta \{(\Delta I_{\mathrm{HR}}/I_{0}{)}_{\lambda_{1}}/(\Delta I_{\mathrm{HR}}/I_{0}{)}_{\lambda_{2}}\}$, where α and β depend on a, b, c, and d. With the assumption that $\Delta I_{\mathrm{HR}}$ is small compared with $I_{0}$ and that there are only two wavelengths, it can be shown that this method is the same as the SD method presented in this work. In fact, Eq. (2) is a combination of Eq. (1), considering two wavelengths, and Beer’s law. This reveals that a, b, c and d all depend on the extinction coefficients of oxy-hemoglobin concentration ($\left[{\mathrm{HbO}}_{2}\right]$) and deoxy-hemoglobin concentration ([Hb]), whereas b and d also depends on ⟨L⟩ at both wavelengths.^28^ Further, in the linearized version, α and β also depend on these extinction coefficients and the ⟨L⟩s. With traditional pulse oximetry these constants are found through empirical calibration while in this work, they are determined using known extinction coefficients and the ⟨L⟩s are assumed from the MC model. Therefore, the difference between traditional pulse oximetry and SD is in calibration, with calibration being empirical for traditional pulse oximetry and the results in this work being un-calibrated as they are based on assumed ⟨L⟩s. Considering this, a majority of results for SD in this work apply to traditional pulse oximetry including, the sensitivity maps, the effect of [M], and the variation across and within subjects (but not the absolute value of ${\mathrm{SpO}}_{2}$).

The second measurement type considered here is DR.^22^^,^^23^ DR is defined as the geometric mean of the ratio between I measurements at a long and short source-detector distance. Changes in the natural logarithm of this geometric mean can be converted to $\Delta \mu_{a}$ using a similar form as Eq. (1)

$$\Delta \mu_{a,\mathrm{DR}}=-\frac{\ln[\sqrt{\frac{I_{1B}I_{2A}}{I_{1A}I_{2B}}}]-\ln[\sqrt{\frac{I_{1B,0}I_{2A,0}}{I_{1A,0}I_{2B,0}}}[}{\overset{¯}{\Delta \langle L\rangle }},$$ (3)

where the subscript DR signifies that the quantity is an effective recovered $\Delta \mu_{a}$ from the DR measurement type. In this case [i.e., Eq. (3)], the proportionality constant is the negative inverse of the average difference in total optical path length ($\overset{¯}{\Delta \langle L\rangle }$) between short and long source-detector distances instead of the negative inverse of ⟨L⟩ as in Eq. (1). This, average difference in total optical path length ($\overset{¯}{\Delta \langle L\rangle }$) will be defined later in Eq. (7).

In either case, a measurement of $\Delta \mu_{a}$ at two or more wavelengths can be converted to a change in oxy-hemoglobin concentration ($\Delta [{\mathrm{HbO}}_{2}]$) and a change in deoxy-hemoglobin concentration (Δ[Hb]) using Beer’s law and their known extinction coefficients.^29^ For this work, we considered four wavelengths with values of 690, 730, 800, and 830 nm.

### 2.3 Monte-Carlo Model and Simulations

The MC model and simulations in this work used the voxel-based Monte-Carlo eXtreme (MCX; rev0313d4 v2020)^30^ called from MATrix LABoratory (MATLAB; rev9.14.0.2286388 v2023a). These simulations were run on a desktop computer with Linux Mint 21.1, an AMD Ryzen 9 7950X3D, 128 GB of the main memory, and an NVIDIA GeForce RTX 4090 with 24 GB of graphic memory.

Two types of MC simulations were run for two different purposes. The first utilized a coarse voxel size and focused on detecting photons at the detector positions to determine the average partial optical path length in the region i ($\left\langle \ell_{i}\right\rangle$), where the different regions (is) were associated with different tissue types. This MC to find $\langle \ell_{i}\rangle s$ launched $10^{9}$ photons and used a voxel size of 0.25×0.25×0.25  mm. The second MC type utilized a fine voxel size with the goal of generating a high-resolution fluence rate (Φ) spatial distribution. This MC type launched $10\times 10^{9}$ photons and used a voxel size of 25×25×25  μm. The two MC types utilized a time range of 0 to 10 ns with only one-time bin so that the results are representative of continuous-wave (CW) methods. In addition, all MC simulations were run three separate times with different random seeds to determine the repeatability of the results.

#### 2.3.1 Finger model

**Fig. 2:**
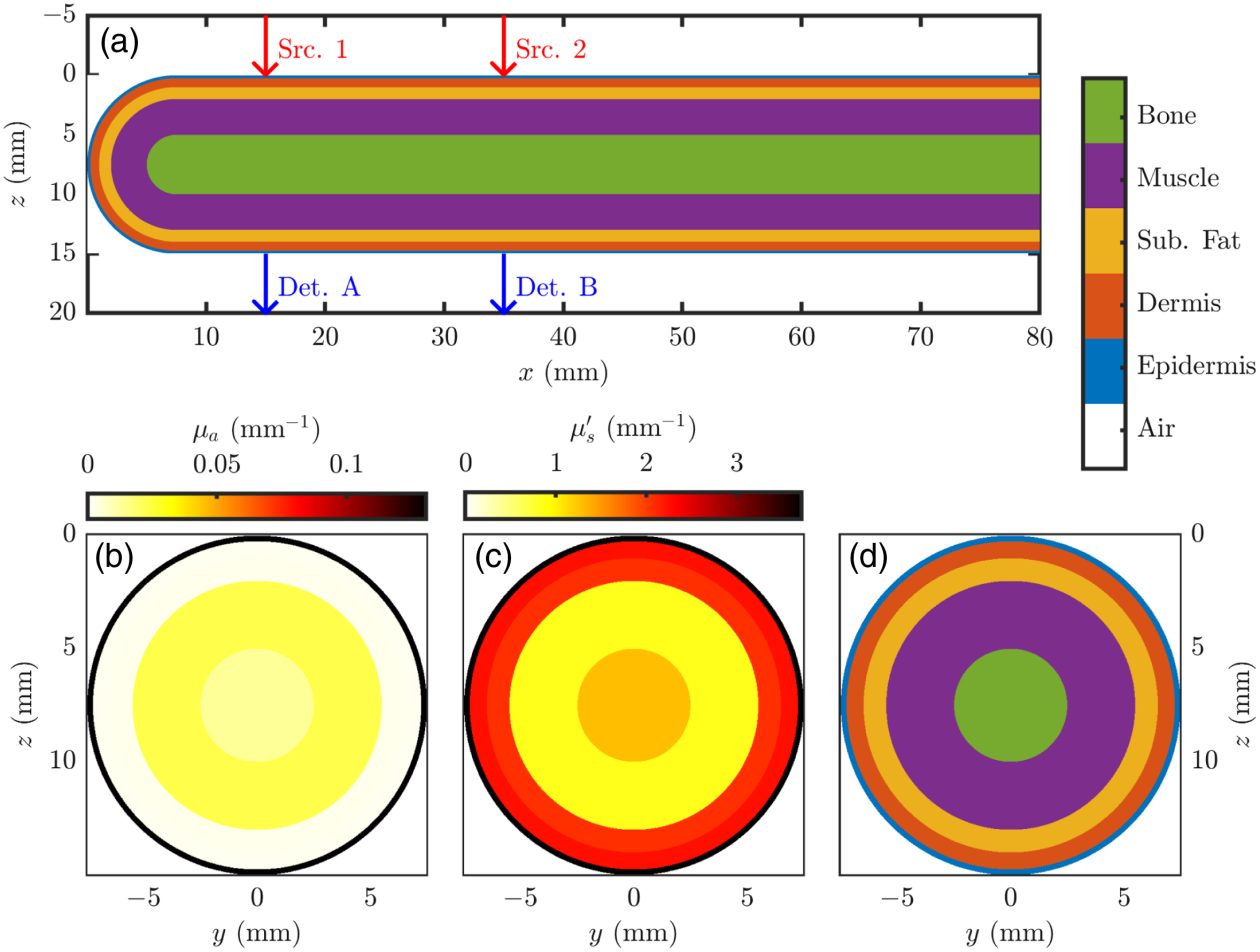
MC finger model. The model consists of a cylinder 80-mm long and 15 mm in diameter with a 15-mm diameter hemisphere representing the fingertip. Source (Src.) 1 is placed at (15x^)  mm, Src. 2 at (35x^)  mm, detector (Det.) A at (15x^+15z^)  mm, and Det. B at (35x^+15z^)  mm. (a) xz slice at y=0  mm of the finger model. (b)–(d) yz slice at x=40  mm. (b) Map of the absorption coefficient ($\mu_{a}$) for the lowest volume fraction of melanosomes ([M]) case ([M]=0.013) and the 800-nm wavelength. (c) Map of the reduced scattering coefficient ($\mu_{s}^{\prime }$) for the 800-nm wavelength. (d) Slice of the finger model with the same color scale as panel (a).

The finger was modeled as an 80-mm long and 15-mm diameter cylinder with a hemisphere at one end to represent the finger tip [Figs. 1(a) and 2]. The outside medium was modeled as air with an index of refraction (n) of 1, and the n mismatch between tissue and air was considered at all boundaries. As shown in Fig. 2, the coordinate system considered the x-axis along the length of the cylinder, the y-axis along the width, and the z-axis along the height (i.e., nail to pad). The origin was placed with x=0  mm at the tip of the hemisphere (i.e., the finger tip), y=0  mm at the center of the cylinder, and z=0  mm at the top of the cylinder (i.e., the plane that the sources are incident upon).

Given this coordinate system, source 1 was placed at (15x^)  mm, source 2 at (35x^)  mm, detector A at (15x^+15z^)  mm, and detector B at (35x^+15z^)  mm (Fig. 2). For the first MC type to find $\langle \ell_{i}\rangle s$, the detectors had radii of 1.5 mm so that the detection area was the surface defined by the intersection of a sphere of this radius centered at the detector coordinate and the surface of the cylinder.

Five tissue types were modeled as shells concentric with both the cylinder and hemisphere allowing each tissue type to be characterized by a layer thickness (Δr; Table 1). These tissue types were the epidermis, dermis, subcutaneous fat (sub. fat), muscle, and bone. Each tissue type was modeled with unique wavelength-dependent optical properties, as described in Tables 1 and 2. The MC simulations were run for each of the four wavelengths (i.e., 690, 730, 800, and 830 nm). Furthermore, a range of [M] was also considered (Table 2). The first MC type to find $\left\langle \ell_{i}\right\rangle$, considered [M] in the range of 0.013 to 0.430 linearly spaced over 1000 values. The second MC to find Φ, considered four values for [M] of 0.013, 0.152, 0.291, and 0.430. For the most part, chromophore concentrations and optical properties were assigned according to Refs. 31 and 32. Tables 1 and 2 specify each specific reference pertinent to each tissue type.

**Table 1:** Modeled optical properties and chromophore concentrations in various tissue regions.

Tissue	λ (nm)	$\mu_{a}$ (${\mathrm{mm}}^{-1}$)	$\mu_{s}^{\prime }$ (${\mathrm{mm}}^{-1}$)	g	n	$\left[{\mathrm{HbO}}_{2}\right]$ (μM)	[Hb] (μM)	[W] ($L/L_{\mathrm{tis}}$)	[L] ($L/L_{\mathrm{tis}}$)	Δr^a^ (mm)
Epidermis^31^^–^^37^	690	^b^	4.3	0.90	1.48	0.0	0.0	0.20	0.00	0.25
	730	^b^	4.1	0.91	1.48					
	800	^b^	3.7	0.92	1.48					
	830	^b^	3.6	0.92	1.48					
Dermis^31^^–^^34^^,^^36^^,^^38^	690	0.0018	2.6	0.90	1.39	1.8	2.9	0.65	0.00	0.75
	730	0.0022	2.5	0.91	1.39					
	800	0.0023	2.2	0.92	1.39					
	830	0.0029	2.2	0.92	1.39					
Sub. Fat^31^^–^^33^^,^^36^^,^^39^^,^^40^	690	0.0023	2.4	0.98	1.49	9.5	3.0	0.11	0.69	1.00
	730	0.0022	2.2	0.98	1.49					
	800	0.0028	2.1	0.98	1.49					
	830	0.0035	2.0	0.98	1.49					
Muscle^31^^,^^32^^,^^36^^,^^41^^–^^43^	690	0.025	1.0	0.95	1.37	75	42	0.80	0.00	3.00
	730	0.019	0.9	0.95	1.36					
	800	0.023	0.8	0.95	1.36					
	830	0.026	0.8	0.95	1.36					
Bone^31^^,^^32^^,^^36^^,^^43^^–^^48^	690	0.0082	1.4	0.94	1.45	61	8.8	0.32	0.00	2.50
	730	0.0083	1.3	0.94	1.45					
	800	0.014	1.3	0.94	1.45					
	830	0.016	1.3	0.94	1.45					

Symbols: Optical wavelength (λ), absorption coefficient ($\mu_{a}$), reduced scattering coefficient ($\mu_{s}^{\prime }$), scattering anisotropy (g), index of refraction (n), oxy-hemoglobin concentration ($\left[{\mathrm{HbO}}_{2}\right]$), deoxy-hemoglobin concentration ([Hb]), water volume fraction ([W]), and lipid volume fraction ([L])

a For the bone, Δr represents the radius, and for other tissues, Δr is the radial thickness.

b See Table 2.

**Table 2:** Modeled absorption coefficient ($\mu_{a}$) for various volume fractions of melanosomes ([M]).^31^^,^^32^^,^^35^^,^^38^^,^^49^^–^^52^

λ (nm)	$\mu_{a}$ (${\mathrm{mm}}^{-1}$)			
	[M]=0.013	[M]=0.152	[M]=0.291	[M]=0.430
690	0.22	2.6	4.9	7.2
730	0.18	2.1	4.0	5.9
800	0.13	1.5	2.9	4.3
830	0.12	1.3	2.6	3.8

Symbols: Optical wavelength (λ), absorption coefficient ($\mu_{a}$), and volume fraction of melanosomes ([M])

#### 2.3.2 Calculation of path lengths and sensitivities

##### Monte-Carlo for partial path lengths

The first MC type was run using a coarse voxel size with the goal of determining the average partial optical path length in region i ($\left\langle \ell_{i}\right\rangle$) (i.e., epidermis, dermis, subcutaneous fat, muscle, and bone) and the average total optical path length (⟨L⟩) for each source-detector pair [a separate MC was run for each source and wavelength, such that $\langle \ell_{i}\rangle s$ and ⟨L⟩s were found for each source-detector pair (i.e., 1A, 1B, 2A, and 2B) and each wavelength (i.e., 690 nm, 730 nm, 800 nm, and 830 nm)]. These MCs were repeated three times to determine uncertainties in the results). This MC was run white [i.e., with zero absorption coefficient ($\mu_{a}$)], and the $\mu_{a}$ of the various tissue types was applied post-runtime. For each detector (i.e., A or B), the partial optical path length (ℓ) spent in each type of tissue by each detected photon was saved. For a particular source-detector pair and wavelength, these ℓs were indexed by tissue region (i) and photon number (γ) so that we have $\ell_{i,\gamma }$.

To take into account the $\mu_{a}$ of the different tissue regions ($\mu_{a,i}$) post-runtime, each individual photon weight ($w_{\gamma }$) was calculated. This was done by scaling the photon $w_{\gamma }$ using the Beer-Lambert law as follows:

$$w_{\gamma }=\overset{N_{\text{regions}}}{\underset{i}{\prod }}e^{-\mu_{a,i}\ell_{i,\gamma }}.$$ (4)

Then, these photon weights ($w_{\gamma }s$) were used to calculate the weighted average of ℓs and yield the average partial optical path length in region i ($\left\langle \ell_{i}\right\rangle$) and the average total optical path length (⟨L⟩) as follows:

$$\langle \ell_{i}\rangle =\frac{\overset{N_{\text{detected}}}{\underset{\gamma }{\sum }}w_{\gamma }\ell_{i,\gamma }}{\overset{N_{\text{detected}}}{\underset{\gamma }{\sum }}w_{\gamma }},$$ (5)

$$\langle L\rangle =\overset{N_{\text{regions}}}{\underset{i}{\sum }}\langle \ell_{i}\rangle ,$$ (6)

where the i subscript on $\left\langle \ell_{i}\right\rangle$ represents the tissue region. Unique values of $\left\langle \ell_{i}\right\rangle$ and ⟨L⟩ were found for each source-detector pair and each wavelength.

⟨L⟩ is the needed proportionality constant in Eq. (1) to convert SD I data to $\Delta \mu_{a}$. For DR, we need the $\overset{¯}{\Delta \langle L\rangle }$ in Eq. (3) to convert DR I data to $\Delta \mu_{a}$. To find $\overset{¯}{\Delta \langle L\rangle }$, we consider the ⟨L⟩ for each source-detector pair (i.e., 1A, 1B, 2A, and 2B), and $\overset{¯}{\Delta \langle L\rangle }$ is calculated as follows:

$$\overset{¯}{\Delta \langle L\rangle }=\frac{\left({\left\langle L\right\rangle }_{1B}-{\left\langle L\right\rangle }_{1A})+({\left\langle L\right\rangle }_{2A}-{\left\langle L\right\rangle }_{2B}\right)}{2}.$$ (7)

Finally, the last output obtained from this first MC type was the S for each tissue region. In the SD case, S for tissue region i is calculated as a ratio of $\left\langle \ell_{i}\right\rangle$ to ⟨L⟩ as follows:^27^^,^^53^

$$S_{\mathrm{SD},i}=\frac{\left\langle \ell_{i}\right\rangle }{\left\langle L\right\rangle }.$$ (8)

For the DR case, S for tissue region i is given by the ratio of the average difference in $\langle \ell_{i}\rangle s$ to the average difference in ⟨L⟩s^27^^,^^53^

$$S_{\mathrm{DR},i}=\frac{\left(\langle \ell_{i}\rangle_{1B}-\langle \ell_{i}\rangle_{1A})+(\langle \ell_{i}\rangle_{2A}-\langle \ell_{i}\rangle_{2B}\right)}{\left(\langle L\rangle_{1B}-\langle L\rangle_{1A})+(\langle L\rangle_{2A}-\langle L\rangle_{2B}\right)}.$$ (9)

In both the SD and DR cases, $S_{i}$ is interpreted as the ratio of the recovered effective $\Delta \mu_{a}$ from a measurement [i.e., from Eq. (1) or Eq. (3)] and a true local $\Delta \mu_{a,i}$ in the tissue region i. This concept is expressed by the following equation:^27^^,^^53^

$$S_{i}=\frac{\Delta \mu_{a,\text{recovered}}}{\Delta \mu_{a,i}}.$$ (10)

Furthermore, these definitions lead to the following property of $S_{i}s$:^27^^,^^53^

$$\sum_{i}^{N_{\mathrm{regions}}}S_{i}=1,$$ (11)

which means that a homogeneous $\Delta \mu_{a}$ (i.e., $\Delta \mu_{a,i}s$ are equal regardless of i) will result in a measured effective recovered $\Delta \mu_{a}$ that is equal to the true homogeneous perturbation.

##### Monte-Carlo for fluence rate distribution

The second MC type was run with a fine voxel size with the goal of determining the spatial distributions of the Φ and then calculating a high-resolution spatial map of S (The first MC type only yielded S for the five tissue regions, whereas the second MC type aims to find S for each voxel to create a spatial map). The outputs of this MC type were the fluence rate (Φ) normalized by source power distributions for a pencil beam placed at each source or detector location. The Φ distribution from a pencil beam at the detector locations was found by mirroring the Φ distributions from the source locations about the plane defined by z=7.5  mm. This approach of mirroring to find Φ from the detectors is possible due to the symmetry in the modeled geometry (Fig. 2).

Φ distributions were used to find S distributions based on a method similar to the adjoint method.^54^ First, to motivate the adjoint method we write the average partial optical path length in voxel j ($\left\langle \ell_{j}\right\rangle$) with voxel volume (V) in terms of a Φ and reflectances (Rs)^53^

$$\langle \ell_{j}\rangle =\frac{\Phi [{\overset{\to }{r}}_{\mathrm{src}}\to {\overset{\to }{r}}_{j}]R[{\overset{\to }{r}}_{j}\to {\overset{\to }{r}}_{\det}]}{R[{\overset{\to }{r}}_{\mathrm{src}}\to {\overset{\to }{r}}_{\det}]}V.$$ (12)

The arguments of Φ or R in Eq. (12) specify the position vectors ($\vec{r}s$) of the voxel field point (j), the source (src), or the detector (det). The direction of light transport is specified by the arrows in the argument (→). Next, we approximate the Rs with Φs and apply the reciprocity relation $\Phi [{\overset{\to }{r}}_{j}\to {\overset{\to }{r}}_{\det}]=(n_{\det}^{2}/n_{j}^{2})\Phi [{\overset{\to }{r}}_{\det}\to {\overset{\to }{r}}_{j}]$ which accounts for the n at the detector (When applying the adjoint method the n at the detector is the n of the medium just below the detector because these voxels below the detector are used to determine the detected Φ) and voxel.^55^ Considering that V, $R[{\overset{\to }{r}}_{\mathrm{src}}\to {\overset{\to }{r}}_{\det}]$, and $n_{\det}$ are constants that do not depend on voxel position, we lump them together into the constant β and rewrite an approximation of Eq. (12)

$$\langle \ell_{j}\rangle ≊\beta \frac{\Phi [{\overset{\to }{r}}_{\mathrm{src}}\to {\overset{\to }{r}}_{j}]\Phi [{\overset{\to }{r}}_{\det}\to {\overset{\to }{r}}_{j}]}{n_{j}^{2}}.$$ (13)

To find β, we can apply Eq. (6) and use the ⟨L⟩ found from the first MC type

$$\beta =\frac{\left\langle L\right\rangle }{\overset{N_{\text{voxels}}}{\underset{j}{\sum }}\frac{\Phi [{\overset{\to }{r}}_{\mathrm{src}}\to {\overset{\to }{r}}_{j}]\Phi [{\overset{\to }{r}}_{\det}\to {\overset{\to }{r}}_{j}]}{n_{j}^{2}}}$$ (14)

for each source-detector pair. After obtaining β using the ⟨L⟩ found from the first MC type, we can use Eq. (13) to find an approximation of $\left\langle \ell_{j}\right\rangle$ for each voxel. Finally, Eqs. (8) and (9) are used to find $S_{j}$ for each voxel and measurement type to create high-resolution spatial maps of S.

#### 2.3.3 Simulation of pulsatile hemodynamics and recovered pulsatile saturation

Using the sensitivity to local absorption change (S) for each tissue region, we can model hemodynamic oscillations (i.e., from cardiac pulsation) in each tissue region and then simulate the associated recovered $\Delta \mu_{a}$. To this aim, we used phasors which describe oscillations at a single frequency as a complex number, whose modulus is the amplitude and argument is the phase of the osculation.^56^ For this work, phasors were used to represent hemodynamic oscillations with a given amplitude and phase at a given frequency (i.e., the heart rate in this case).^57^ Phasors are helpful when considering linear combinations of oscillations, as is the case when combining pulsatile oscillation contributions from different tissues. Further, hemodynamic phasors can provide insight into relationships between blood volume and blood flow oscillations in the tissue.^53^^,^^58^ These hemodynamic phasors for $\left[{\mathrm{HbO}}_{2}\right]$ and [Hb] were modeled in each tissue (e.g., ${\overset{˜}{\left[{\mathrm{HbO}}_{2}\right]}}_{\text{Muscle}}$ for the phasor of the oscillation in $\left[{\mathrm{HbO}}_{2}\right]$ in the muscle). Then, these phasors were converted to $\mu_{a}$ phasors in each tissue at each of the four wavelengths using Beer’s law and known extinction coefficients (e.g., ${\overset{˜}{\mu_{a}}}_{,\text{Muscle}}(830\;\mathrm{nm})$ for the phasor of the oscillation in $\mu_{a}$ at 830 nm in the muscle).^29^^,^^53^ Next, for each wavelength, the recovered $\mu_{a}$ phasors were found by a linear combination of the $\mu_{a}$ phasors in each tissue region weighed by the $S_{i}$ in the tissue region i [Eq. (10)] as follows:^53^

$${\overset{˜}{\mu_{a}}}_{,\text{Recovered}}=\overset{n_{\text{regions}}}{\underset{i=1}{\sum }}S_{i}{\overset{˜}{\mu_{a}}}_{,i}.$$ (15)

These simulated recovered $\mu_{a}$ phasors at four wavelengths are then converted to recovered $\left[{\mathrm{HbO}}_{2}\right]$ and [Hb] phasors, again using Beer’s law.^29^^,^^53^ Finally, the ${\mathrm{SpO}}_{2}$ is obtained from these $\left[{\mathrm{HbO}}_{2}\right]$ and [Hb] phasors ($\tilde{\left[{\mathrm{HbO}}_{2}\right]}$ and $\tilde{\left[\mathrm{Hb}\right]}$) as follows:

$${\mathrm{SpO}}_{2}=\frac{\left|\overset{˜}{\left[{\mathrm{HbO}}_{2}\right]}\right|}{\left|\overset{˜}{\left[{\mathrm{HbO}}_{2}\right]}|+|\overset{˜}{\left[\mathrm{Hb}\right]}\right|}.$$ (16)

We observe that for ${\mathrm{SpO}}_{2}$ in Eq. (16) to be representative of the blood oxygen saturation of a volume oscillating vasculature compartment (i.e., as with ${\mathrm{SaO}}_{2}$), the phasors $\tilde{\left[{\mathrm{HbO}}_{2}\right]}$ and $\tilde{\left[\mathrm{Hb}\right]}$ must be in phase with each other.^58^ If this is not the case, one needs to apply a correction to take into account the phase difference between $\tilde{\left[{\mathrm{HbO}}_{2}\right]}$ and $\tilde{\left[\mathrm{Hb}\right]}$.^57^

For the simulations, the recovered ${\mathrm{SpO}}_{2}$ represents what would be recovered given the modeled hemodynamic phasors in each tissue region. This can be done for either measurement type (i.e., SD or DR), and in the case of SD, for each source-detector pair (i.e., 1A, 1B, 2A, and 2B). Note that Eq. (16) not only applies to simulations but also represents how we calculate ${\mathrm{SpO}}_{2}$ in general for this work, including for the *in vivo* data. We further emphasize that this method of recovering ${\mathrm{SpO}}_{2}$ with Eq. (16) from $\left[{\mathrm{HbO}}_{2}\right]$ and [Hb] phasors individually does not account for the phase relationship between $\left[{\mathrm{HbO}}_{2}\right]$ and [Hb].

### 2.4 *In Vivo* Measurements

#### 2.4.1 Recovery of pulsatile saturation

For the *in vivo* measurements, we used the finger clip probe shown in Fig. 1(b) and collected the intensity (I) between each source and detector (i.e., 1A, 1B, 2A, and 2B) and for each wavelength (i.e., 690, 730, 800, and 830 nm) (The protocol for these measurements is described in Sec. 2.4.3). The inner surface of the probe was made of black silicone to ensure that no light exiting the tissue could be reflected and reenter the tissue. The first step in the analysis of these *in vivo* data was to convert these measured I to $\Delta \mu_{a}$ for each wavelength and measurement type [i.e., SD (For this work, the SD pair focused upon was 1A.) and DR]. This conversion was done using Eqs. (1) and (3), assuming the ⟨L⟩s or $\overset{¯}{\Delta \langle L\rangle }s$ obtained from the first MC type described in Sec. 2.3.2. Built into this assumption are the finger’s optical properties and geometry, including the value of [M] in the epidermis. Therefore, the *in vivo* data may be analyzed with different assumed values for [M] (${\left[M\right]}_{ass}$), to investigate the effect of such assumption.

From the temporal traces of $\Delta \mu_{a}$ at the four wavelengths, the temporal $\Delta [{\mathrm{HbO}}_{2}]s$ and Δ[Hb]s were found using Beer’s law.^29^ Working with $\Delta [{\mathrm{HbO}}_{2}]$ from DR data, we next find the heart frequency (i.e., heart rate), which is then assumed to be the same for all measurement types within one dataset. To find this frequency, the data were first de-trended so that the first and last temporal points took the value of 0  μM. Then, a high-pass filter with a cutoff of 50/60 Hz (i.e., 0.83 Hz) was applied to the signal. Next, the filtered temporal data is transformed into the Fourier domain using a fast Fourier transform (FFT) with a Nuttall Blackman-Harris window.^59^ Considering only frequencies below 2.5 Hz, the peak in the Fourier domain with the highest amplitude was identified. To find the peak centroid, we considered the peak extending from the first minimum below the frequency of maximum amplitude to the first minimum above the said maximum amplitude frequency point. The centroid frequency was calculated as a weighted average frequency, weighted by the amplitude of each frequency point which comprised the peak; this centroid was taken as the heart frequency.

Knowing the heart frequency, the temporal signals of $\Delta [{\mathrm{HbO}}_{2}]$ and Δ[Hb] associated with each data type are band-pass filtered about it. The band-pass filter utilized a central frequency equal to the heart frequency and a bandwidth of 10 mHz (this small bandwidth filter is needed to apply the Hilbert transform in the next step). At this point, the signals are analyzed in two different ways: an FFT to determine amplitudes and a Hilbert transform to determine phases. For the amplitudes, an FFT of the band-passed signals was taken and the amplitude was determined as the integral of this Fourier spectrum from the beginning to the end of the heart-frequency peak defined as before. The integral bounds are larger than the band-pass bandwidth so that the entire peak is included in the interval. We will write the amplitudes found in this way as $\left|\tilde{\left[{\mathrm{HbO}}_{2}\right]}\right|$ and $\left|\tilde{\left[\mathrm{Hb}\right]}\right|$. To determine the phase of $\Delta [{\mathrm{HbO}}_{2}]$ and Δ[Hb] at the heart frequency, we employed the Hilbert transform on the band-passed signals. The phase reference was considered to be the phase of the change in total hemoglobin concentration (i.e., $\Delta [{\mathrm{HbO}}_{2}]+\Delta [\mathrm{Hb}]$) measured by DR. We will write the phases found in this way as $∠\overset{˜}{\left[{\mathrm{HbO}}_{2}\right]}$ and $∠\tilde{\left[\mathrm{Hb}\right]}$.

Finally, we utilized the amplitudes, $\left|\tilde{\left[{\mathrm{HbO}}_{2}\right]}\right|$ and $\left|\tilde{\left[\mathrm{Hb}\right]}\right|$, to calculate ${\mathrm{SpO}}_{2}$ using Eq. (16). These recovered ${\mathrm{SpO}}_{2}$ values are obtained for each measurement type but also for different assumed values of [M]. Therefore, in this work, we investigate how assumed values of [M] affect the recovered ${\mathrm{SpO}}_{2}$. Further, we will utilize the recovered $∠\tilde{\left[{\mathrm{HbO}}_{2}\right]}$ and $∠\tilde{\left[\mathrm{Hb}\right]}$ to aid in discussing how phase differences between $\left[{\mathrm{HbO}}_{2}\right]$ and [Hb] oscillations affect the recovered ${\mathrm{SpO}}_{2}$.

#### 2.4.2 Determination of skin tone

To connect the values of [M] in the MC models with the *in vivo* data, we have quantified the skin tone of the human subjects we measured. For the determination of skin tone, we utilized the Monk scale.^60^ The Monk scale consists of 10 swatches which represent a wide range of skin tones, with Monk 1 being the lightest and Monk 10 being the darkest.

To determine each subject’s skin tone, a photo of the back of their hand was taken with a Canon EOS Rebel T3i digital camera. For these photos, the stock 18- to 55-mm lens was used set at the 55-mm focal length. The camera took a photo from ∼1  m distance with a 60-W incandescent light source at almost the same location as the camera. The incandescent light source was the only light source in the room when the photo was taken. The subject’s hand was placed on a white background and the camera focused on the back of the hand. Camera settings were set to full manual and were the same for each subject. The primary camera settings were an ISO of 100, an aperture of f/5, and an exposure time of 0.3 s. We would like to note that these settings are considered to slightly overexpose the scene; however, no part of the images was saturated.

**Fig. 3:**
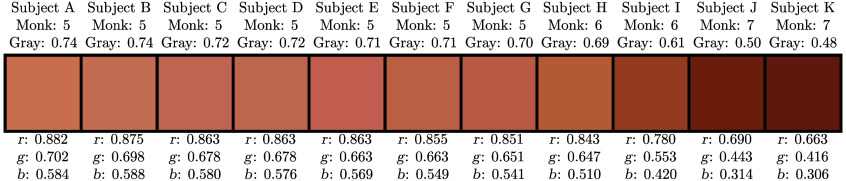
Color swatches of each subject’s skin tone obtained from a photo of the back of the subject’s hand. For each subject, the Monk scale^60^ value, gray-scale value (using Rec.ITU-R BT.601-7), and normalized red-green-blue (RGB) values are also reported.

Each photo was saved in the Canon raw image format (CR2) and was 5184×3456  px in size. A crop of 500×500  px around the center of the back of the subject’s hand was used for further analysis. This cropped image was averaged to find the average red-green-blue (RGB) color value. These color swatches of the RGB value for each subject are shown in Fig. 3. At this point, we note that these RGB color values are comparable with each other due to the control over camera setup and parameters, but the values would be difficult to compare with color values independently measured by other photographers.

To quantify the subject’s Monk scale value, we compared the subject’s RGB skin tone value with values derived from photos of Monk scale color swatches. The Monk scale swatches were printed on white paper using a Canon iR ADV C5250 color printer. The printed swatch palette was then photographed with the same settings and at the same location as the photos of the subject’s hand. Finally, the determination of the Monk scale value for each subject was found by minimizing the difference between the subject’s RGB value and the RGB values for the Monk color swatches. We again note that these values are comparable between subjects in this paper but would be difficult to compare with other photos or Monk scale values determined in different ways. These Monk scale values are reported in Fig. 3 and Table 3.

**Table 3:** Subject information.

Subject	Age	Sex at birth	Monk skin tone^60^ (See Fig. 3)	Race	Ethnicity	Majority ancestral region
A	26	Female	5	Multi	Hispanic	North-America
B	31	Female	5	White	Non-Hispanic	Southern-Europe
C	28	Male	5	Asian	Non-Hispanic	Southeast-Asia
D	30	Male	5	White	Non-Hispanic	Northern- and Eastern- Europe
E	23	Female	5	White	Non-Hispanic	Western-Europe
F	25	Male	5	Asian	Non-Hispanic	East-Asia
G	59	Male	5	White	Non-Hispanic	Southern-Europe
H	23	Female	6	Multi	Hispanic	North-America & Southeast-Asia
I	27	Female	6	Asian	Non-Hispanic	Southern-India
J	25	Male	7	Black	Non-Hispanic	Africa
K	29	Male	7	Asian	Non-Hispanic	Southern-India

#### 2.4.3 Protocol and subjects

For all *in vivo* experiments, LD light was delivered to the clip probe [Fig. 1(b)] and detected from the clip probe using optical fiber bundles. We believe that the use of LDs in our proposed method is important because it alleviates possible complications introduced by sources with a large spectral bandwidth (such as LEDs). This negates the need to consider the shift of a broad spectral peak when the light passes through the melanin-rich epidermis.^18^^,^^19^ The use of LDs is feasible in diffuse optics because only the tight spectral bandwidth feature is of importance. Therefore, low-power and low-cost LDs may be used, and a diffuse filter may even be added to the LD to alleviate safety concerns. The source fibers delivered light from or to an ISS Imagent V2 Frequency-Domain (FD) NIRS instrument. The FD NIRS instrument used LDs at wavelengths of 690, 730, 800, and 830 nm, a 140.625 MHz modulation frequency, and a 9.93 Hz sample rate. Four wavelengths were used instead of the two typical for pulse oximetry to diminish any inaccuracies arising from the choice of wavelength or spectral characteristics of the sources. The amplitude of the FD NIRS data was taken as a close approximation of CW I in the context of this work.

Eleven healthy human subjects (i.e., labeled A to K) were recruited and consented according to the Tufts University Institutional Review Board protocol for this study. The subjects’ age, sex, Monk scale value,^60^ race, ethnicity, and majority ancestral region are reported in Table 3. We report racial and ethnic information for each subject to give context to their skin tones beyond our quantification on the Monk scale. In addition, subjects are ordered A to K in the order of their swatch gray-scale value; this ordering corresponds to Monk scale value ordering but with more precision (Fig. 3). Gray-scale values were calculated using the rgb2gray() MATLAB function which utilizes the Rec.ITU-R BT.601-7 standard. This order is used to better aid the interpretation of the data in terms of skin tone.

For the experimental protocol, each subject was asked to sit in a chair with their left hand placed on a stool in front of them, so that their hand was approximately at the height of their chest. All of the subjects in this work reported being right-hand dominant. Before each experiment, we ensured that the subject’s hand was warm, using a space heater when necessary. The subject placed their left-index finger in the clip probe shown in Fig. 1(b); all subjects in this study reported being right-handed. The source side of the probe corresponded to the subject’s finger top (i.e., the nail/knuckle side) and the detector side corresponded to the finger bottom (i.e., the pad side). Furthermore, their fingers were placed within the clip probe such that source 1 was just behind the nail (i.e., source light was not transmitted through the nail). Finally, 3 min of NIRS data consisting of the I between each source and detector was collected for each subject. These data were converted to $\Delta [{\mathrm{HbO}}_{2}]$ and Δ[Hb] then to ${\mathrm{SpO}}_{2}$ for both SD and DR as described in Sec. 2.4.1.

## 3 Results

### 3.1 Measurement Path Lengths and Sensitivities from Monte-Carlo Simulations

**Fig. 4:**
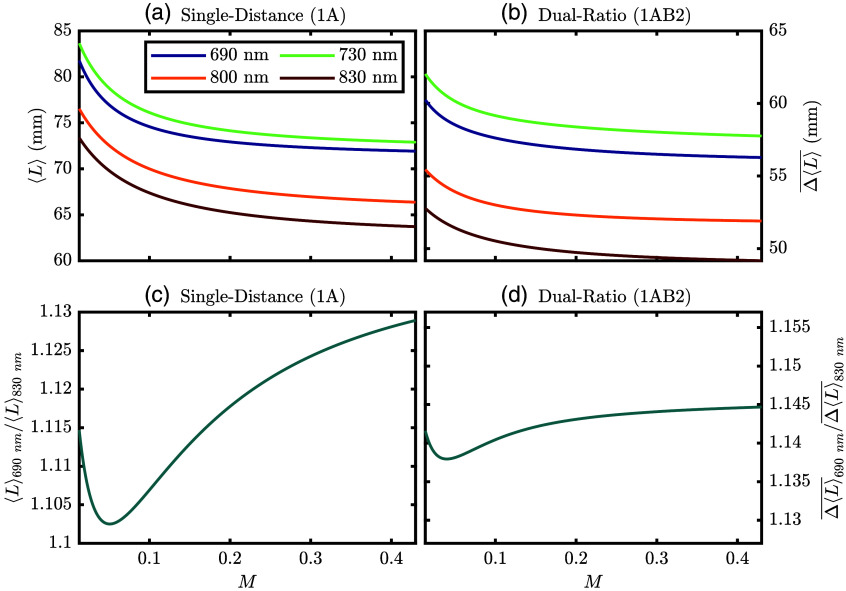
MC-derived average total optical path lengths (⟨L⟩s) and the average difference in total optical path lengths ($\overset{¯}{\Delta \langle L\rangle }s$) for different optical wavelengths (λs) as a function of volume fraction of melanosomes ([M]). Also, see Table 4. (a) ⟨L⟩ for the SD pair formed by source 1 and detector A (Fig. 2). (b) $\overset{¯}{\Delta \langle L\rangle }$ for the DR set [Fig. 2; Eq. (7)]. (c) Ratio of ⟨L⟩ at 690 nm over ⟨L⟩ at 830 nm. (d) Ratio of $\overset{¯}{\Delta \langle L\rangle }$ at 690 nm over ⟨L⟩ at 830 nm. Note: Subplots (c) and (d) are on the same scale but not on the same range.

First, we present the results from the MC simulations. Figure 4 and Table 4 show the ⟨L⟩ from the first MC type. The ⟨L⟩ and the $\overset{¯}{\Delta \langle L\rangle }$ are important because they are the proportionality constants needed to convert optical data (i.e., I) to $\Delta \mu_{a}s$ [Eqs. (1) and (3)]. Focusing on Fig. 4(a), we see that ⟨L⟩ decreases with increasing [M] in the epidermis, with a stronger decrease at lower values of [M]. This is likely due to the photons that take longer paths around the circumference of the finger having a lower probability of surviving when they encounter a more absorbing epidermis. Next, we may examine how [M] affects $\overset{¯}{\Delta \langle L\rangle }$ in Fig. 4(b). Here, much like ⟨L⟩, $\overset{¯}{\Delta \langle L\rangle }$ also decreases with increasing [M]. However, let us examine how much ⟨L⟩ or $\bar{\Delta \langle L\rangle }$ changes from the lowest to highest [M] (i.e., from [M]=0.013 to [M]=0.430) at 830 nm using the values in Table 4. In this case, ⟨L⟩ changes by –13% and $\bar{\Delta \langle L\rangle }$ changes by –7.0%; we remind that ⟨L⟩ is needed for SD measurements and $\overset{¯}{\Delta \langle L\rangle }$ is needed for DR measurements. To consider the case with higher pigmentation on the dorsal (top/nail side) versus palmar (bottom/pad side), as is often the case anatomically, we conducted an additional simulation. We considered an epidermis [M] of 0.291 on the dorsal side (z≤7.5  mm) and 0.152 on the palmar side (z>7.5  mm) of the finger. This simulation resulted in values for ⟨L⟩ and $\overset{¯}{\Delta \langle L\rangle }$ in-between the values for the homogeneous [M]=0.013 and [M]=0.430 simulations (Table 4). Thus, we opted to only consider and present models with homogeneous [M] in the epidermis, as a simulation with bilateral [M] would not produce significantly different results.

**Table 4:** MC-derived average total optical path lengths (⟨L⟩s) and average difference in total optical path lengths ($\overset{¯}{\Delta \langle L\rangle }s$); also see Fig. 4.

[M]	λ (nm)	${\left\langle L\right\rangle }_{1A}$ (mm)	${\left\langle L\right\rangle }_{1B}$ (mm)	${\left\langle L\right\rangle }_{2A}$ (mm)	${\left\langle L\right\rangle }_{2B}$ (mm)	${\overset{¯}{\Delta \langle L\rangle }}_{1\mathrm{AB}2}$ (mm)
0.013	690	81.79 ± 0.02	142.2 ± 0.2	141.9 ± 0.3	81.82 ± 0.02	60.2 ± 0.2
	830	73.38 ± 0.01	126.03 ± 0.06	126.3 ± 0.1	73.37 ± 0.02	52.78 ± 0.07
0.430	690	71.92 ± 0.04	128 ± 1	128.0 ± 0.3	71.98 ± 0.06	56.3 ± 0.6
	830	63.70 ± 0.03	112.5 ± 0.6	113.3 ± 0.3	63.76 ± 0.03	49.1 ± 0.3

Acronyms: Volume fraction of melanosomes ([M]), optical wavelength (λ), average total optical path length (⟨L⟩), average difference in total optical path length ($\overset{¯}{\Delta \langle L\rangle }$)

Note: Values represent the mean of three MCs with different random seeds and the error is half the range of the three MCs

When interpreting these values for ⟨L⟩ and $\overset{¯}{\Delta \langle L\rangle }$ in terms of their relevance toward recovering ${\mathrm{SpO}}_{2}$, it is important to consider all wavelengths. This is done in Figs. 4(c) and 4(d) where the ratio of ⟨L⟩ or $\overset{¯}{\Delta \langle L\rangle }$ between 690 and 830 nm is plotted. In the case of ⟨L⟩, this ratio would be proportional to the calibration factor applied in traditional ${\mathrm{SpO}}_{2}$ measurements. This is because traditional ${\mathrm{SpO}}_{2}$ uses the ratio of the normalized pulsatile amplitude at red and infrared wavelengths.^3^ Here, we can also compare how much these ratios change from low to high [M]. The ratio of ⟨L⟩s changes from 1.11 to 1.13 for [M]=0.013 to [M]=0.430, respectively, whereas the ratio of $\overset{¯}{\Delta \langle L\rangle }s$ changes from 1.142 to 1.145 in the same range of [M]. This is a change of 1.3% for the ratio of ⟨L⟩ and a change of 0.27% for $\bar{\Delta \langle L\rangle }$. This suggests that the calibration factor for DR measurements would likely be less sensitive to [M] than the factor for SD measurements. However, in either case, these results suggest that these calibration factors change only by a few percent across [M] values.

**Fig. 5:**
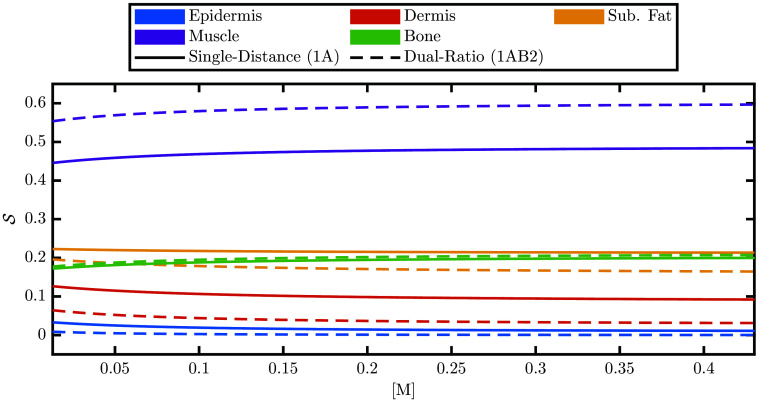
The sensitivity to local absorption change (S) at 800 nm for the five different modeled tissues (Fig. 2) as a function of the volume fraction of melanosomes ([M]). Solid lines show the S for the SD measurement type and dashed lines for the DR measurement type.

Figure 5 shows the S in different tissue regions for various values of [M] and for the two measurement types, SD or DR. In general, both measurement types have the highest S to muscle and lowest S to epidermis. Comparing SD and DR, we see that DR is less sensitive to dynamics in superficial tissues such as the dermis and epidermis but more sensitive to dynamics in deep tissues such as the muscle when compared with SD. For example, the epidermal S is 3.30% for SD and 0.87% for DS when [M]=0.013, and 1.11% for SD and 0.03% for DS when [M]=0.430. In addition, looking at the dependence on [M], we see that sensitivity to local absorption change (S) is lost in superficial tissues, whereas deep tissues gain S at higher [M] values. However, these dependencies on [M] are weak with only a change of a few percent across the full range of [M] values considered.

**Fig. 6:**
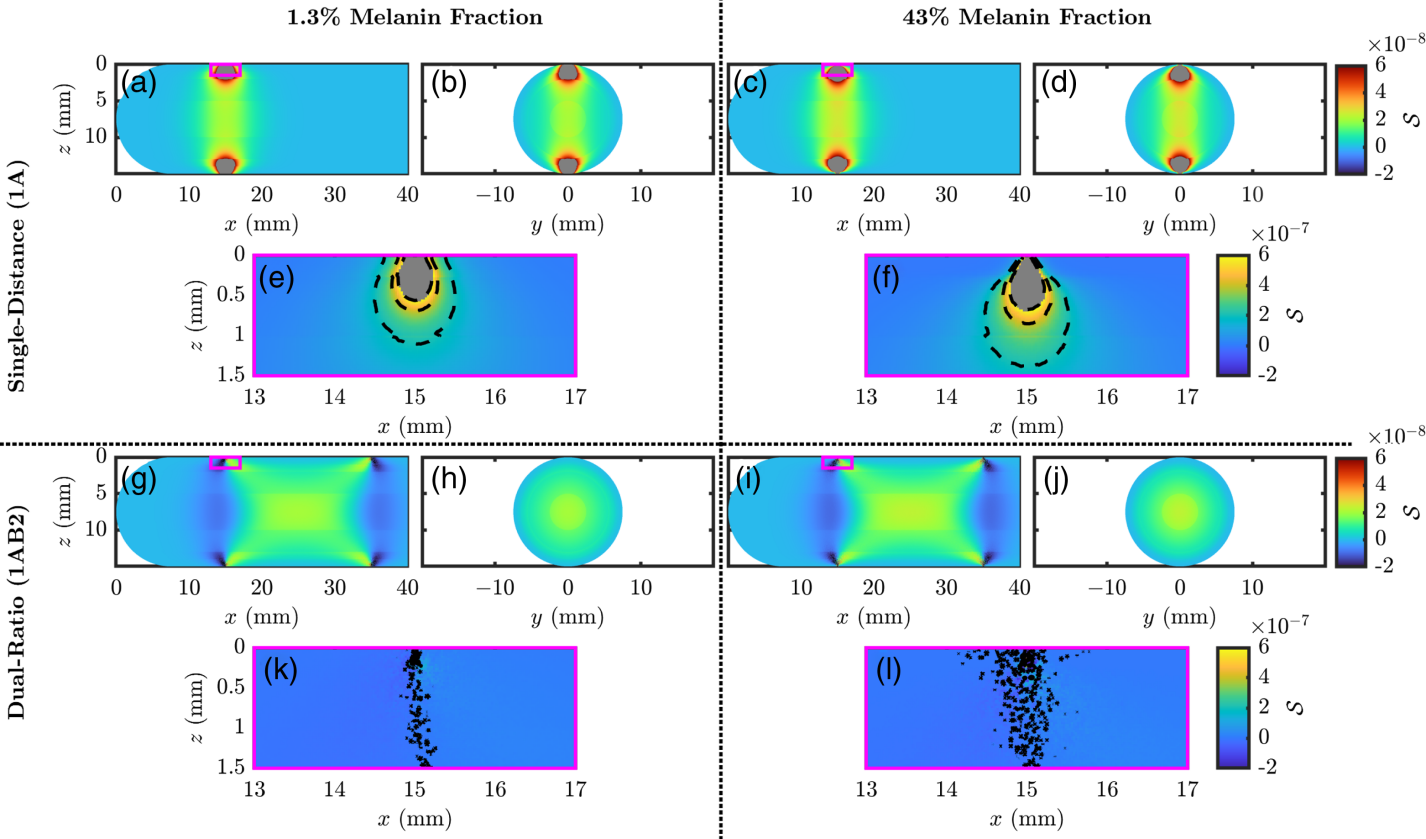
Spatial maps of the sensitivity to local absorption change (S) at 800 nm for the SD) (a)–f) or DR (g)–(l) measurement types and volume fraction of melanosomes ([M]) of 0.013 (a), (b), (e), (g), (h), and (k) or 0.430 (c), (d), (f), (i), (j), and (l). Panels (e), (f), (k), and (l) show a zoomed view of the region indicated by the magenta box in panels (a), (c), (g), and (i), respectively.

Finally, for this section, we shall look at Fig. 6 which contains the spatial S maps for the two measurement types (i.e., SD and DR) and two volume fractions of melanosomes ([M]) values of 0.013 and 0.430. In general, these S maps show a similar story to that of Fig. 5, with higher values of [M] resulting in a decrease in superficial S and an increase in deep or centralized S. We can also look at the S very close to the optodes in Figs. 6(e), 6(f), 6(k), and 6(l). As seen between Figs. 6(e) and 6(f), there is a reduction of S to the epidermis when a high [M] is considered, which results in a deepening of the bulb of high S beneath the optodes for SD measurements. However, in Figs. 6(k) and 6(l), we see little to no S to superficial tissue for the DR measurement type regardless of [M]. Note that Figs. 6(k) and 6(l) show black iso-lines that have a speckled nature, which is because many values in the zoomed map are near zero and noise in the MC is beginning to influence the iso-line shape.

In summary, both Figs. 5 and 6 help inform where the measurements of effective $\Delta \mu_{a}$ come from and show that different measurement types (i.e., SD and DR) have different sensitivities to different tissue regions. Therefore, if tissue hemodynamics are heterogeneous, the partial volume effect governing the recovery of effective $\Delta \mu_{a}$ will result in different recovered hemodynamics for different measurement types. For this reason, in further sections, we consider hemodynamic models that contain different oscillations in different tissues to investigate the effects of this partial volume effect.

### 3.2 Recovered Pulsatile Saturation from *In Vivo* Data

**Fig. 7:**
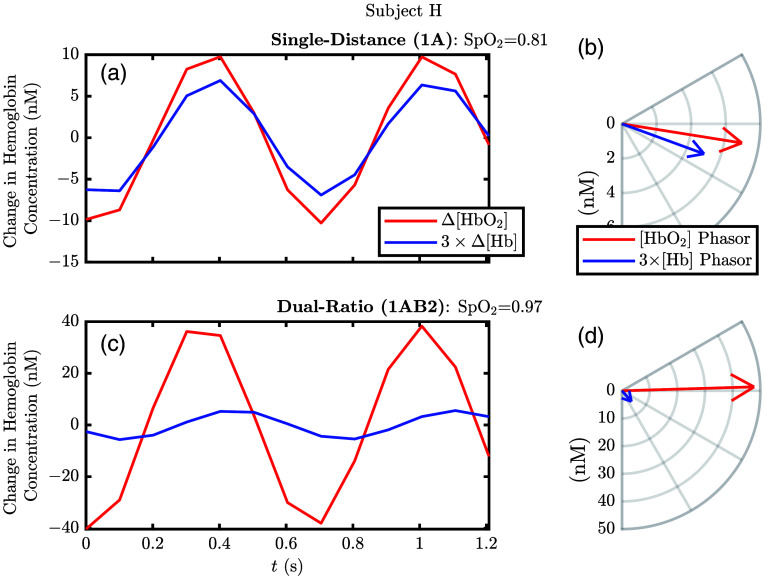
Example hemodynamics measured by SD and DR for subject H. (a) Folding average for two periods of the band-passed temporal traces of change in oxy-hemoglobin concentration ($\Delta [{\mathrm{HbO}}_{2}]$) and change in deoxy-hemoglobin concentration (Δ[Hb]) measured by SD 1A (Fig. 1). (b) Phasors for (a) which have the values: $\overset{˜}{\left[{\mathrm{HbO}}_{2}\right]}=(7.0∠-9.1\;\deg)\;\mathrm{nM}$ and $\overset{˜}{\left[\mathrm{Hb}\right]}=(1.7∠-20.1\;\deg)\;\mathrm{nM}$. (c) Same as panel (a) but measured by DR instead. (d) Phasors for (c) which have the values: $\overset{˜}{\left[{\mathrm{HbO}}_{2}\right]}=(48∠1.7\;\deg)\;\mathrm{nM}$ and $\overset{˜}{\left[\mathrm{Hb}\right]}=(1.7∠-48.6\;\deg)\;\mathrm{nM}$. Note: The assumed volume fraction of melanosomes ([M]) for this example is 0.013, and the phase reference is $\Delta [{\mathrm{HbO}}_{2}]+\Delta [\mathrm{Hb}]$ measured by DR; see Sec. 2.4.1 for further details on analysis. Note: See footnote k on page 30 regarding recovered pulsatile blood oxygen saturation (${\mathrm{SpO}}_{2}$).

Now, we present the results from the *in vivo* experiments. Figure 7 shows an example of recovered hemodynamic folding average traces and phasors from subject H. Figures 7(a) and 7(c) contain folding average traces over two periods of the heart frequency for the band-pass filtered (i.e., with a central frequency of the heart frequency) $\Delta [{\mathrm{HbO}}_{2}]$ and Δ[Hb] temporal traces from SD and DR measurement types, respectively, whereas Figs. 7(d) and 7(d) show the corresponding hemodynamic phasors for the oscillations in Figs. 7(a) and 7(c), respectively. Notice that the amplitudes of these oscillations are on the order of nM due to the small bandwidth of the band-pass filter resulting in little remaining power in the Fourier spectrum (for comparison the noise floor is on the order of 0.1 nM in the Fourier spectrum making the heart-frequency peak aignal-to-noise ratio (SNR) on the order of 10). For this dataset, the SNR (i.e., considering the amplitude of the cardiac pulsation to be signal) of the raw data was ∼10 for SD long, SD short, and DR at 830 nm. Interestingly, the SNR at long and short SDs was similar due to a higher signal amplitude at the long distance.

In this work, we do not consider the phase relationship of $\Delta [{\mathrm{HbO}}_{2}]$ and Δ[Hb] when calculating ${\mathrm{SpO}}_{2}$. However, we show the phasors including their phase relationship in Fig. 7 to enable discussion of this consideration and possible future work because it is not considered in Eq. (16). Continuing to consider the example in Fig. 7, for subject H, we see the ${\mathrm{SpO}}_{2}$ recovered from DR was 97%, whereas from SD, it was 81% (we consider these ${\mathrm{SpO}}_{2}$ measurements uncalibrated because they do not represent ${\mathrm{SaO}}_{2}$ due to different partial volume effects, and typical ${\mathrm{SpO}}_{2}$ techniques would effectively apply a calibration factor to these values to recover a ${\mathrm{SaO}}_{2}$ surrogate). A further observation is that for both SD and DR the [Hb] phasor has a more negative phase relative to the $\left[{\mathrm{HbO}}_{2}\right]$ phasor. This may suggest that the true tissue hemodynamics are a mixture of blood volume (BV) (i.e., in phase) and blood flow (BF) (i.e., out of phase) oscillations at the heart frequency, not solely BV oscillations as is required for ${\mathrm{SpO}}_{2}$ measurements.

**Fig. 8:**
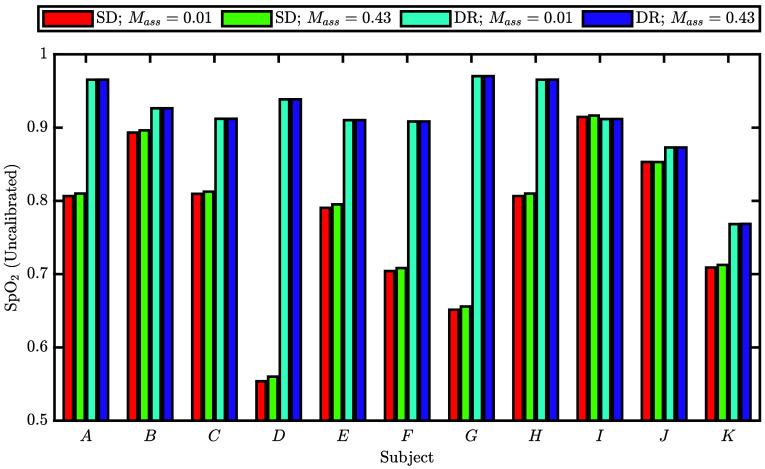
Recovered pulsatile blood oxygen saturation (${\mathrm{SpO}}_{2}$) (we consider these ${\mathrm{SpO}}_{2}$ measurements uncalibrated because they do not represent ${\mathrm{SaO}}_{2}$ due to different partial volume effects, and typical ${\mathrm{SpO}}_{2}$ techniques would effectively apply a calibration factor to these values to recover an ${\mathrm{SaO}}_{2}$ surrogate) *in vivo* from either SD or DR measurements using an assumed volume fraction of melanosomes ([M]) of 0.013 or 0.430. Subjects are ordered from light to dark skin tones according to Sec. 2.4.2 and Fig. 3.

We now move from the example data set in Fig. 7 to a summary of the recovered ${\mathrm{SpO}}_{2}$ for all subjects in Fig. 8. Here, we show the recovered ${\mathrm{SpO}}_{2}$ with SD and DR for assumed [M] values of 0.013 and 0.430. In all cases, except subject I, the recovered ${\mathrm{SpO}}_{2}$ from DR was higher than the recovered ${\mathrm{SpO}}_{2}$ from SD; for subject I, the recovered ${\mathrm{SpO}}_{2}$ with the two measurement types was close to equal. Furthermore, examining the dependence on the assumed value of [M], we see that a higher [M] increases the recovered ${\mathrm{SpO}}_{2}$ for SD by a small amount, on the order of 0.5%, but it has no noticeable effect on the recovered ${\mathrm{SpO}}_{2}$ with DR. Because Fig. 8 shows no substantial difference between the ${\mathrm{SpO}}_{2}$ recovered using different assumed values of [M], the assumed values of [M] have little impact on the analysis methods presented here (Sec. 2.4.1), especially in the case of DR.

We may also examine Fig. 8 in terms of skin tone dependence. As a reminder, we have ordered the subjects A to K in order from lightest to darkest skin tone according to the methods in Sec. 2.4.2 and Fig. 3. The SD measurements appear to have little to no dependence on skin tone, though the values of recovered ${\mathrm{SpO}}_{2}$ vary greatly with a minimum of 55% for subject D to a maximum of 91% for subject I. By contrast, the variation of recovered ${\mathrm{SpO}}_{2}$ values for DR across subjects is much less, with a minimum of 77% for subject k and a maximum of 97% for subject G. We also point out that Fig. 8 may show a dependence on skin tone for DR measurements if we look at the recovered ${\mathrm{SpO}}_{2}$ values from subject H to subject K. This may suggest an effect that results in a lower recovered ${\mathrm{SpO}}_{2}$ for the darker skin when using DR. This would represent a negative bias which is at odds with results for conventional pulse oximetry in the literature.^16^^,^^17^ These lower ${\mathrm{SpO}}_{2}$ recovered by DR, particularly for subjects J and K, are not corrected by assuming relevant [M] (i.e., there is no noticeable difference between assuming [M] of 0.013 or 0.430 for any subject). We caution that this dependence observed in subjects H to K is tenuous due to the small number of subjects and the observed variations in ${\mathrm{SpO}}_{2}$ across all subjects. We only point out this dependence as a possible point of discussion that will be further investigated in future work, which will also consider the phase differences between $\Delta [{\mathrm{HbO}}_{2}]$ and Δ[Hb] (Fig. 7).

### 3.3 Recovered Saturation from Monte-Carlo Simulations Informed by *In Vivo* Results

As described in Sec. 2.3.3, we may simulate either SD or DR measurements for different tissue hemodynamics and [M] values based on the MC model. Furthermore, we can simulate measurements for given hemodynamics and a value for [M] but analyze the data with a different assumed value of [M] to investigate the effect of such an assumption. Of course, these simulations are with the caveat of being within the context of the MC finger model in this work.

**Fig. 9:**
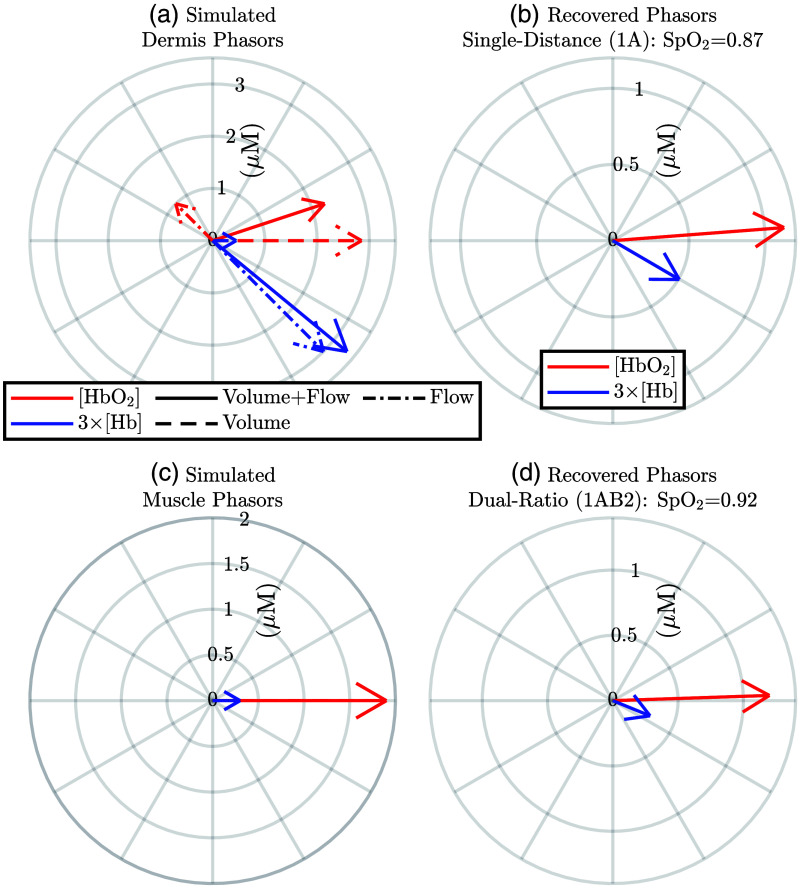
Simulation of tissue oxy-hemoglobin concentration ($\left[{\mathrm{HbO}}_{2}\right]$) and deoxy-hemoglobin concentration ([Hb]) phasors leading to recovered phasors and pulsatile blood oxygen saturation (${\mathrm{SpO}}_{2}$) measured by SD or DR. Hemodynamics are only in the dermis (BV and BF) and muscle (only BV). BV oscillations have a saturation of 95%. Volume fraction of melanosomes ([M]) is modeled as 0.013. (a) Dermis phasors: (2.26∠18.3  deg)  μM for $\left[{\mathrm{HbO}}_{2}\right]$ and (1.11∠−39.5  deg)  μM for [Hb]. (b) SD recovered phasors: (1.13∠4.4  deg)  μM for $\left[{\mathrm{HbO}}_{2}\right]$ and (0.17∠−30.3  deg)  μM for [Hb]. (c) Muscle phasors: (1.90∠0  deg)  μM for $\left[{\mathrm{HbO}}_{2}\right]$ and (0.10∠0  deg)  μM for [Hb]. (d) DR recovered phasors: (1.20∠1.9  deg)  μM for $\left[{\mathrm{HbO}}_{2}\right]$ and (0.10∠−22.1  deg)  μM for [Hb].

One consistent result of the *in vivo* measurements reported in Fig. 8 was that ${\mathrm{SpO}}_{2}$ recovered by DR was greater than the one recovered by SD. This may be explained by different hemodynamics in different tissues wherein SD and DR have a different sensitivity to local absorption change (S) making the partial volume effect come into play (Figs. 5 and 6). We report a simulation of hemodynamic phasors at the heart frequency that recreates this consistent result. To that end, we may model hemodynamics as combinations of BV and BF, with $\left[{\mathrm{HbO}}_{2}\right]$ and [Hb] phasors being in-phase if driven by BV oscillations and out of phase if driven by BF oscillations. Further, the relative amplitudes of the arterial BV components of the $\left[{\mathrm{HbO}}_{2}\right]$ and [Hb] phasors are ${\mathrm{SaO}}_{2}$. For BF components, the amplitudes of $\left[{\mathrm{HbO}}_{2}\right]$ and [Hb] phasors are equal. Considering this, we modeled hemodynamics as a combination of BF and BV with an ${\mathrm{SaO}}_{2}$ of 95%. Oscillations were assumed to be in the dermis and muscle only, with only the dermis having a BF contribution. In the dermis, the BF components were (1∠135  deg)  μM for the $\left[{\mathrm{HbO}}_{2}\right]$ phasor and (1∠−45  deg)  μM for the [Hb] phasor, whereas BV components were (2.85∠0  deg)  μM for the $\left[{\mathrm{HbO}}_{2}\right]$ phasor and (0.15∠0  deg)  μM for the [Hb] phasor [Fig. 9(a)]. Meanwhile, for the muscle, the BV components were (1.90∠0  deg)  μM for the $\left[{\mathrm{HbO}}_{2}\right]$ phasor and (0.10∠0  deg)  μM for the [Hb] phasor [Fig. 9(c)]. Given this simulation and the model, the recovered phasors are shown in Figs. 9(b) and 9(d) which recreate the greater ${\mathrm{SpO}}_{2}$ recovered by DR which was observed *in vivo*. We also note that the simulation in Fig. 9 qualitatively recreates the phase relationships between $\left[{\mathrm{HbO}}_{2}\right]$ and [Hb] phasors shown in the example dataset in Fig. 7, though we do not put much weight on this agreement because it is with one example subject.

We can extend these simulations by investigating the assumed value of [M] for ${\mathrm{SpO}}_{2}$. For this, we consider three different hemodynamic simulations (i.e., two simulations in addition to the one already described). One hemodynamic simulation is the one in Fig. 9 which recreated some aspects of the *in vivo* results and considers a simulation of BV and BF in the dermis and only BV in the muscle. The first additional simulation assumed a homogeneous BV oscillation and ${\mathrm{SaO}}_{2}$ of 0.95% in all tissues, with no BF oscillations anywhere. That is an $\left[{\mathrm{HbO}}_{2}\right]$ phasor of (0.95∠0  deg)  μM and an [Hb] phasor of (0.05∠0  deg)  μM in all tissues. This homogeneous case will only show the effect of different assumed values of [M]. The second additional simulation is similar to the first, with only BV oscillations, but now only in the dermis and muscle and no hemodynamics at the heart frequency in the other tissues. This case also considered an $\left[{\mathrm{HbO}}_{2}\right]$ phasor of (0.95∠0  deg)  μM and an [Hb] phasor of (0.05∠0  deg)  μM, but now only in the dermis and muscle.

**Fig. 10:**
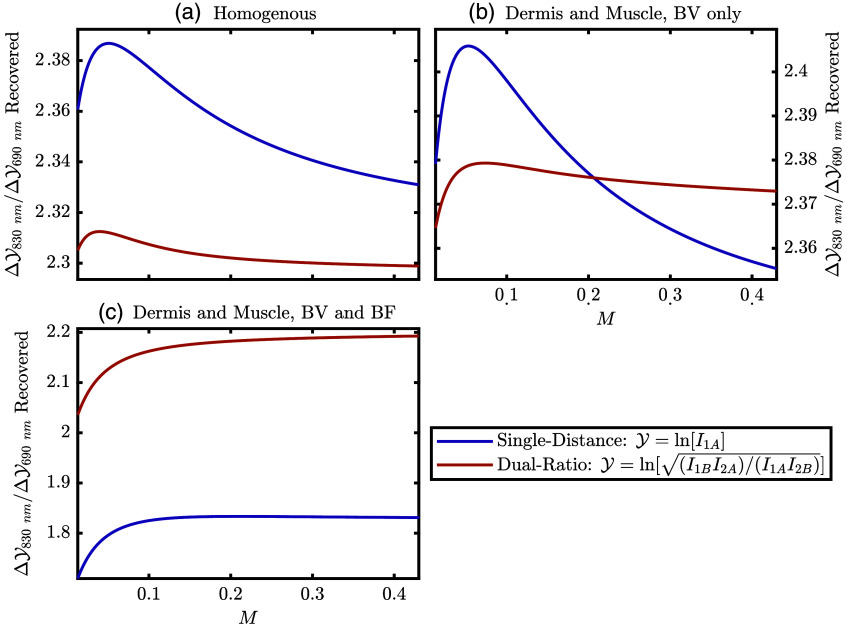
Simulated ratios of the changes in optical data (Y) at 830 nm and 690 nm as a function of modeled [M]. Changes in Y are the numerators of Eq. (1) or Eq. (3) which are also written in the legend for either SD or DR. (a) Simulation with homogeneous BV oscillations in the whole tissue with phasor values of (0.95∠0  deg)  μM for oxy-hemoglobin concentration ($\left[{\mathrm{HbO}}_{2}\right]$) and (0.05∠0  deg)  μM for deoxy-hemoglobin concentration ([Hb]). (b) Simulation with BV oscillations only in the dermis and muscle again with phasor values of (0.95∠0  deg)  μM for $\left[{\mathrm{HbO}}_{2}\right]$ and (0.05∠0  deg)  μM for [Hb]. (c) Simulation with BF and BV oscillations in the dermis and only BV in the muscle. This is the same simulation as Fig. 9, and the simulated phasor values may be found there.

Figure 10 shows simulated recovered data for the three hemodynamic simulations presented in a similar way to the data collected for traditional ${\mathrm{SpO}}_{2}$ methods, as ratios of pulsatile components of change in optical data (ΔY) at two wavelengths. In Fig. 10, the ratio of changes in ΔY at 830 and 690 nm are plotted versus [M]. For SD and DR changes in ΔY are given by the numerators of the right-hand side of Eqs. (1) and (3), respectively. The three panels of Fig. 10 represent the three simulations. One will note that the recovered data for both SD and DR does depend on [M], but the dependence on [M] is stronger for SD in the two simulations which assume no BF oscillations (i.e., the first and second).

**Fig. 11:**
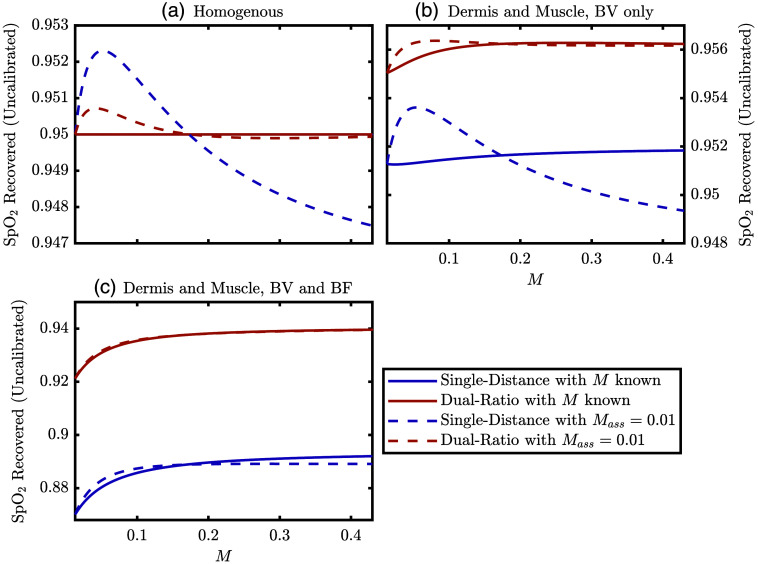
Simulated recovered pulsatile blood oxygen saturation (${\mathrm{SpO}}_{2}$) for SD or DR. Solid lines are the case where the true volume fraction of melanosomes ([M]) is known and used to recover ${\mathrm{SpO}}_{2}$, whereas dashed lines assume a value for [M] of 0.013. For all simulations, the true arterial blood oxygen saturation (${\mathrm{SaO}}_{2}$) from BV) oscillations is 95%. (a) Simulation with homogeneous BV oscillations in the whole tissue with phasor values of (0.95∠0  deg)  μM for oxy-hemoglobin concentration ($\left[{\mathrm{HbO}}_{2}\right]$) and (0.05∠0  deg)  μM for deoxy-hemoglobin concentration ([Hb]). (b) Simulation with BV oscillations only in the dermis and muscle again with phasor values of (0.95∠0  deg)  μM for $\left[{\mathrm{HbO}}_{2}\right]$ and (0.05∠0  deg)  μM for [Hb]. (c) Simulation with BF and BV oscillations in the dermis and only BV in the muscle. This is the same simulation as Fig. 9, and the simulated phasor values may be found there.

Finally, we come to Fig. 11 which conveys the same information as Fig. 10 but with the added information of what the recovered ${\mathrm{SpO}}_{2}$ would be for different cases of assumed [M]. Figure 11 considers all four wavelengths, but Fig. 10 only considers 690 and 830 nm. The three simulations described above are shown in Fig. 11, and the dashed lines represent simulated data analyzed with an assumed [M] of 0.013. In all three simulations, the true ${\mathrm{SaO}}_{2}$ (i.e., the saturation of the BV oscillation) is 95%. The true [M] in the simulation (i.e., the one used to generate the forward data) corresponds to the value on the x-axis, and solid lines are recovered ${\mathrm{SpO}}_{2}$ using this true [M] value. First, let us look at Fig. 11(a), which shows the simulation with homogeneous BV oscillations in all the tissue regions. The solid lines in Fig. 11(a) match the simulated ${\mathrm{SaO}}_{2}$ and recover a ${\mathrm{SpO}}_{2}$ of 95%, which verifies the validity of the methods in this work. Note that the solid lines in Fig. 11(a) are coincident. Further, the deviation of the dashed line from the solid line shows the effect of assuming an incorrect value of [M] without any partial-volume effects confounding the simulation. From this, we see that the recovered ${\mathrm{SpO}}_{2}$ from SD deviates from the true value by less than 0.25%, whereas the recovered ${\mathrm{SpO}}_{2}$ from DR deviates by less than 0.1%. Moving to the second simulation in Fig. 11(b), which only considers BV oscillations that are in the dermis and muscle, we see a similar story to Fig. 11(a) but with a systematic shift for the SD or DR recovered ${\mathrm{SpO}}_{2}$. In both cases, ${\mathrm{SpO}}_{2}$ overestimates the simulated ${\mathrm{SaO}}_{2}$, with DR overestimating it more, with the difference at about 0.6%. These shifts of the solid lines in Fig. 11(b) result from partial volume effects. Finally, we move to the last simulation in Fig. 11(c), which is the same as Fig. 9. In this case, there is a large difference between ${\mathrm{SpO}}_{2}$ recovered by SD and DR. This result matches the *in vivo* data in that the value for DR is greater regardless of [M]. In addition, the recovered ${\mathrm{SpO}}_{2}$ varies by about 2% across [M] for both measurement types, and this is not corrected for by knowing the true [M] when analyzing the data (i.e., the solid curve varies more than the difference between the solid and dashed curves). Because the recovered ${\mathrm{SpO}}_{2}$ in Fig. 11(c) increases with increasing [M], this is at odds with the decrease observed between subjects H and K as shown in Fig. 8, further suggesting that this observation from Fig. 8 may not be significant.

Wrapping up these simulations, we can see that the partial volume effect and heterogeneous tissue hemodynamic phasors affect ${\mathrm{SpO}}_{2}$ values recovered by both SD and DR to a greater extent than different values of [M], whether or not the correct value of [M] is known. Furthermore, in the simulation which roughly recreated the *in vivo* results [Fig. 11(c)] DR recovered a ${\mathrm{SpO}}_{2}$ value closer to the modeled ${\mathrm{SaO}}_{2}$. This is consistent with the higher values of ${\mathrm{SpO}}_{2}$ found by DR in the *in vivo* data presented in Fig. 8. But these trends in Fig. 11 show changes in recovered ${\mathrm{SpO}}_{2}$ that are much less than the observed differences in recovered ${\mathrm{SpO}}_{2}$ between different subjects in Fig. 8. These greater differences *in vivo* suggest that the simulations may not be capturing the full picture. We conclude this section by reminding the readers that these results are all within the context of the MC model we used in this work, which assumes a particular anatomy, optical properties, and model of skin tone based on [M].

## 4 Discussion

In this work, we presented a MC model to simulate optical measurements on the human finger and experimental results obtained *in vivo* using the same optical measurement geometry (Fig. 1). We designed the finger MC model based on a simplified geometry of tissue types in a human finger and assumed optical properties based on literature.^31^^,^^32^ Then, we used this model to analyze the *in vivo* data and recover ${\mathrm{SpO}}_{2}$ for two different measurement types (i.e., SD and DR, where SD represents traditional pulse oximetry). The results of the *in vivo* experiment were then used to inform hemodynamic oscillation models enabling a discussion of the differences between measurement types, assumed [M], and heterogeneous tissue hemodynamics. We have chosen this approach to analyze both SD (i.e., representative of traditional pulse oximetry) and DR data using an MC model so that all assumptions are known. This allows for a fair comparison of SD and DR, with any differences between the results of recovered ${\mathrm{SpO}}_{2}$ being meaningful in themselves because we know they have been treated with the same analysis methods. A comparison of this type would not be possible against results obtained using a commercial pulse oximeter.

The MC simulations culminated in the results shown in Fig. 11 for three different hemodynamic models. These models considered hemodynamic oscillations at the heart rate (i.e., heart frequency) in different tissue regions with different phase and amplitude relationships. One particular hemodynamic model was created to roughly match some consistent results obtained in the *in vivo* measurements. By contrast, two other hemodynamic models were used to investigate the interplay between tissue hemodynamic heterogeneity and assumed values of [M]. Overall, the results showed that the assumed value for [M], whether assumed incorrectly or correctly, affected the recovered ${\mathrm{SpO}}_{2}$ on the order of 1%. However, the modeled heterogeneity of tissue hemodynamics significantly affected the recovered ${\mathrm{SpO}}_{2}$; this effect was greater than the effect from assumed [M]. However, we do not know the true variability of the hemodynamics in the subject fingers; thus, this is only one possible explanation of the observed variability in ${\mathrm{SpO}}_{2}$. Due to the magnitude of the variability in ${\mathrm{SpO}}_{2}$, the effect of the skin tone bias may be obscured making it hard to observe with our small subject population. The different results obtained with different measurement types are due to different partial volume effects. These differences between measurement types were observed *in vivo*, pointing to evidence of spatially heterogeneous tissue hemodynamics.

Expounding upon these ideas regarding assumed values of [M] or spatially varying tissue hemodynamics, we can focus on Figs. 4, 10, and 11. Figure 4 represents the proportionality constants recovered from the MC simulations, which govern how changes in the measured optical data are converted to the $\Delta \mu_{a}s$, which are further converted to the $\Delta [{\mathrm{HbO}}_{2}]$ and Δ[Hb], and then finally ${\mathrm{SpO}}_{2}$. Meanwhile, the simulations reported in Fig. 10 show that the measured optical data are affected by different hemodynamic situations and [M] values. Finally, Fig. 11 shows the simulated recovered ${\mathrm{SpO}}_{2}$ for the same hemodynamic situations as in Fig. 10. The lines in Fig. 11 effectively result from a multiplication of the values in Figs. 4(c) and 4(d) and Fig. 10 (this statement is not formally true because Figs. 4(c) and 4(d) and Fig. 10 consider two wavelengths, but Fig. 11 uses all four wavelengths in this work). This relationship, between the values of Figs. 4, 10, and 11, shows the interplay between the measured optical data and assumed calibration constants (obtained from MC in the work) needed to recover $\Delta \mu_{a}$, which is related to ${\mathrm{SpO}}_{2}$ through known extinction coefficients.^29^ Effectively, the curves in Figs. 4(c) and 4(d) are the calibration constants that would need to be found to convert optical data to a measurement proportional to ${\mathrm{SpO}}_{2}$ Therefore, the variation of the values in Figs. 4(c) and 4(d) over [M] shows how these calibration constants may need to change with skin tone. Meanwhile, Figs. 10 and 11 add the consideration of heterogeneous tissue hemodynamic oscillations and how the recovered measurements would change with them.

Looking at all of these considerations together, we see that the recovered ${\mathrm{SpO}}_{2}$ was more influenced by hemodynamic heterogeneity in the way we modeled them than assumed [M]. We justify this considering that because different people will likely have different hemodynamic heterogeneity, we expect the recovered ${\mathrm{SpO}}_{2}$ to vary more across subjects than across assumed [M] values within a subject. This is in fact observed in Fig. 8 wherein the variation across subjects is much greater than the differences obtained by assuming different [M] values. Furthermore, the variation across subjects is greater for SD compared with DR suggesting that DR, at least in part, compensates for the subject differences. We can put these results in the context of Ref. 17 which showed a bias in the ${\mathrm{SpO}}_{2}$ measured on Black versus White patients. In Ref. 17, the variation across patients (i.e., for a given value of ${\mathrm{SaO}}_{2}$) was greater than the observed skin tone bias. This skin tone bias, in Ref. 17, is evident when considering the large patient population which, when averaged, may have suppressed the hemodynamic or physiological differences between the patients making the effect of skin tone apparent. In fact, the variance of the measurements of ${\mathrm{SpO}}_{2}$ in Ref. 17 is larger than the observed magnitude of the skin tone bias, necessitating a large subject population. Further, Ref. 11, a meta-analysis, points out the imprecision of pulse oximetry and arrives at the same conclusion, that the skin tone bias is difficult to observe with statistical confidence. These suggest that the observed bias toward higher ${\mathrm{SpO}}_{2}$ for persons with dark skin may be obscured by the large variability and imprecision of pulse oximetry to do other factors. Our results are in line with this interpretation of the results in Refs. 17 and 11 given that we expect more variation in ${\mathrm{SpO}}_{2}$ from other confounds (such as hemodynamic differences) than from skin tone (i.e., modeled as [M] in this work). However, we caution against the interpretation that the large variability and imprecision of pulse oximetry are the result of hemodynamic heterogeneity. Instead, we are suggesting that this may be a possible cause, which may even exacerbate the skin tone bias in some cases, but more investigation is necessary.

One criticism of traditional ${\mathrm{SpO}}_{2}$ measurements is that the calibration factor is assumed to be the same for all skin tones.^9^^,^^21^ For the results obtained from the models, simulations, and analysis methods reported in this article, we see that this assumption creates small inaccuracies in ${\mathrm{SpO}}_{2}$, on the order of 1%. Instead, the results in this work show that an additional confound on recovered ${\mathrm{SpO}}_{2}$ may be the heterogeneity of tissue hemodynamics or similar confounds.

Beyond the discussion of [M] and tissue hemodynamic heterogeneity, we also explored the differences between measurement types, SD and DR. One consistent result that we see from the *in vivo* data was a higher ${\mathrm{SpO}}_{2}$ recovered by DR compared with SD. More importantly, the ${\mathrm{SpO}}_{2}$ recovered by DR was more consistent across the healthy human subjects, which is in line with these subjects having approximately the same nominal ${\mathrm{SaO}}_{2}$. This result was recreated in a simulation that modeled pulsatile BV in the dermis and muscle tissue, with pulsatile BF only occurring in the dermis. However, these hemodynamic models and the relationship of tissue sensitivities are complex, so we do not claim that this simulation, which recreated the *in vivo* data relationships, is necessarily representative of the actual tissue dynamics. While this case may be possible, other situations may also reproduce our *in vivo* results.

## 5 Conclusion

Our results indicate that optical measurements of ${\mathrm{SpO}}_{2}$ may be dependent on heterogeneity in tissue hemodynamics in addition to the assumed value of [M]. Furthermore, we found that DR measurements recovered greater and more consistent ${\mathrm{SpO}}_{2}$ values than SD measurements (i.e., where SD is representative of traditional pulse oximetry) across various healthy human subjects. These results will further enable discussions about optimal methods to recover ${\mathrm{SpO}}_{2}$ with minimal impact from skin tone. Further, these results show the promise of the DR method for ${\mathrm{SpO}}_{2}$ measurements on the human finger. Future directions include considering the amplitude and phase relationships of pulsatile dynamics of $\Delta [{\mathrm{HbO}}_{2}]$ and Δ[Hb] when recovering ${\mathrm{SpO}}_{2}$, finding ways to better assess and account for different [M]s and skin tones, investigating pulsatile hemodynamic heterogeneity in the finger, considering the number and values of wavelengths used, using of novel measurement methods such as DR, and considering time-resolved optical changes in the time or frequency domain. We hope that this work may represent a jumping-off point for future work on these considerations.

## Data Availability

Applicable supporting code and data are available from the authors upon reasonable request.
